# Supplementary material for: Who Will Be More Egocentric? Age Differences in the Impact of Retrospective Self-Experience on Interpersonal Emotion Intensity Judgment
Source: Behav Sci (Basel). 2024 Apr 4;14(4):299. doi: 10.3390/bs14040299 (PMC11047662; doi:10.3390/bs14040299)
Supplement: Supplementary file 1 [file behavsci-14-00299-s001.zip › behavsci-2901853-supplementary.pdf]

Supplement A

The pilot study—the process of compiling material, rating and screening

The pilot study was used to design the interpersonal emotion scenarios used for the emotion judgment tasks in the formal experiments. The pilot study included (1) interviewing younger and older adults about their daily positive and negative emotions and the corresponding emotional events; (2) compiling the interpersonal emotion scenarios based on the interview results; and (3) screening out the required material pools for formal experiments.

The interview about daily emotional event

Thirty-two younger adults (age: M=23.34; SD=1.28) and 28 older adults (age: M=65.11; SD=3.91) were recruited for interviews. The participants were initially asked to report their daily frequently occurring emotional events and the emotion they felt during that events, and each person reported 3 positive events and 3 negative events. Joy and pride were selected as positive emotions (frequency ratio of older adults: joy 76.25%, pride 20%; frequency ratio of younger adults: joy 63.27%, pride 21.43%), and anger, distress and sadness were selected as negative emotions in the formal experiment (frequency ratio of older adults: anger 8.45%, distress 46.48%, sadness 8.45%; frequency ratio of younger adults: anger 22.22%, distress 37.37%, sadness 14.14%), according to the top frequency among the two groups. Although the frequency of guilt was 16.16% for younger adults, anger, distress and sadness accounted for relatively high proportions in both age groups and were selected to better compare the characteristics of the two age groups. The highly frequent emotional event themes mentioned were used for later scenario material compiling.

Participants were then required to rate the frequency with which their

emotions were biased, perceived and judged by others in daily life (from 1 "never" to 5 "always"). The mean rating score of younger adults was M=2.84 (SD=0.81), and that of older adults was M=2.32 (SD=0.61). Finally, participants were asked to rate the degree of influence of this category of biased emotional judgment events on their life (from 1 "no influence" to 5 "strong influence"). The mean rating score of younger adults was M=2.91 (SD=0.86), and that of older adults was M=2.14 (SD=0.76). According to these results, the frequency and impact of such biased emotional judgment on the lives of younger and older adults are moderate. The frequency and influence of such events are significantly higher for young people than for older people, $t_{frequency}$ (58)=2.97， p<.01; $t_{frequency}$ (58)=3.64, p<0.001. Thus, there may be age differences in the occurrence and subsequent impact of interpersonal bias in emotional judgments on older adults and younger adults.

Compiling the emotion scenario material

After determining the emotion category for the experimental material, the emotional event theme and category were screened out according to the frequency at which they were mentioned in interviews. One or two emotion scenario themes for each emotion category that showed a high frequency in interviews were selected.

For older adults, the selected **joy-related scenario themes** were "interpersonal interaction" (frequency ratio: 32.50%, e.g., visits from children, meeting with friends) and "leisure activities" (frequency ratio: 17.50%, e.g., singing and dancing, playing chess). The selected **pride-related scenario themes** were "personal achievement" (frequency ratio: 11.25%, e.g., work achievement, winning a prize) and "achievement of children" (frequency ratio: 21.25%, e.g., children's graduation, children's job

promotion). The selected **anger-related scenario themes** were “interpersonal relationship” (frequency ratio: 22.53%, e.g., spousal relationship, parent-child relationship) and “man-made accident” (frequency ratio: 23.94%, e.g., theft, fraud). The selected **distress-related scenario themes** were “interpersonal relationship” (frequency ratio: 22.53%, e.g., spousal relationship, parent-child relationship) and “accident caused by self” (frequency ratio: 23.94%, e.g., property loss). The selected **sadness-related scenario themes** were “interpersonal relationship” (frequency ratio: 22.53%, e.g., spousal relationship, parent-child relationship) and “health-related accident” (frequency ratio: 30.99%, e.g., disease in the family, disease in self).

For younger adults, the selected **joy-related scenario themes** were “interpersonal interaction” (frequency ratio: 27.55%, e.g., help from friends, meeting with friends) and “leisure activities” (frequency ratio: 17.50%, e.g., travelling, watching concerts). The selected **pride-related scenario theme** was “personal achievement” (frequency ratio: 35.71%, e.g., obtaining a scholarship, fulfilling a hard task). The selected **anger-related scenario themes** were “interpersonal relationship” (frequency ratio: 27.27%, e.g., romantic relationship, friend relationship) and “man-made accident” (frequency ratio: 19.19%, e.g., property damage, fraud). The selected **distress-related scenario themes** were “study and work” (frequency ratio: 32.32%, e.g., spousal relationship, parent-child relationship) and “accident caused by self” (frequency ratio: 19.19%, e.g., stay up late to complete homework, working overtime). The selected **sadness-related scenario themes** were “study and work” (frequency ratio: 32.32%, e.g., examination failure, contest failure) and “interpersonal relationship” (frequency ratio:

30.99%, e.g., romantic relationship, parent-child relationship).

Then, we compiled the emotion scenarios required for the experiment for each scenario theme and category and both younger and older adults. **All emotional events were compiled using the following standards:**

(1) Scenario frame: **protagonist** + **details 1** (age, gender or other personal characteristics) + **details 2** (lifestyle habits and hobbies, have some correlation with the subsequent events) + **emotional event** (events able to elicit moderate levels of the target emotion)

(2) Number of words: 30-50 words in Chinese

(3) Protagonists' gender and age ensured a certain balance in the overall situation.

An example of a joy-related event scenario is as follows:

Example scenario for older adults: Ms Li, a 70-year-old woman, has been living alone for many years. Her daughter will visit her during the Mid-Autumn Festival.

Example scenario for younger adults: Ming has just started college. None of his former friends live in the same city. Last week, one of his high school classmates visited him.

We compiled 100 emotion scenarios for both younger adults and older adults (20 scenarios for each emotion). These scenarios were rated by a calibration group of recruited participants. Ninety-six younger adults (age: M=2.84; SD=0.81) and 90 older adults (age: M=2.84; SD=0.81) rated the scenarios in terms of **familiarity** (the extent to which you have encountered or seen such incidents in your daily life) and **importance**

(the importance attached to the occurrence and handling of such incidents) on a seven-point Likert scale (from 1 = Not familiar/important at all to 7 = Extremely familiar/important) and then **confirmed** the emotion felt by the protagonist in the scenario for a given emotion category ("yes" or "no") and estimated the emotion intensity (0-100) felt by the protagonist in the scenario for given emotion category.

Emotion scenario material screening and selection

After collecting the rating data of the calibration group, the emotion scenarios were screened to ensure no significant difference between the older group and the younger group in the evaluation of importance and familiarity. And the scenarios would be divided into target scenarios and anchor-generating scenarios. **The screening and selection standard was as follows:**

(1) Scenario screening: emotion scenarios with five or more negative answers were screened out (emotion category confirming with "no").

(2) Scenarios selection: scenarios generating medium-intensity emotion (65-75) were selected for constructing **the target scenario**. Besides, scenarios generating high-intensity emotion (higher than 75) were selected for constructing **anchor-generating scenarios in high-anchor conditions**, and scenarios generating low-intensity emotion (lower than 65) were selected for constructing **anchor-generating scenarios in low-anchor conditions**.

Finally, 66 emotion scenarios from older adults and 70 emotion scenarios from younger adults were used as the final scenario pool for the corresponding experiment. The descriptive information of the mean rating on familiarity and importance in each

age group is shown in Table A. The descriptive information of emotion intensity for the target scenario and anchor-generating scenario has been presented in manuscript Table 1. Finally, 10 target scenarios, 10 anchor-generating scenarios for high-anchor conditions, and 10 anchor-generating scenarios for low-anchor conditions were selected for experimental materials from the scenario pools.

Table S1. Description of familiarity, the importance for young and older adults in positive and negative emotion scenarios

Measures	Emotional valence	Old		Young	
		M	SD	M	SD
Familiarity	Positive emotion	4.85	1.05	4.68	0.94
Familiarity	Negative emotion	4.58	1.31	4.70	1.02
Importance	Positive emotion	4.98	0.78	4.89	0.77
Importance	Negative emotion	4.76	1.07	4.84	0.96

The materials for target scenarios which depict a protagonist encountering a specific emotion-generating event could be directly used in the formal experiment. The materials for anchor-generating scenarios need additional minor revisions to better put participants in the role of the protagonist, and then remind their own experience when reading scenarios. The revision included replacing the protagonist with a second personal pronoun as "you', and fuzzily processing character features, such as career state. The revised example of an anchor-generating scenario is like this: "You have an intimate relationship with your wife, last week your wife was seriously ill in

hospital". The final selected material pool for the formal experiment also ensured that the mean emotion-intensity rating of older and younger groups' materials would not be significantly different. The calibration group of sixty-two older adults (age: M=64.18; SD=4.01) and fifty-five younger adults (age: M=22.62; SD=3.30) rated the emotion intensity on the revised version material for high and low anchor group of anchor-generating scenario. The result indicated that the rated mean emotion intensity of the high anchor-generating scenario is significantly higher than that in the low anchor-generating scenario (M_{high}=80.00 M_{low}=61.22, t (110) =8.46, p<0.001, MD=18.78), which verified the validity of manipulation on anchor.

The mean emotion intensity rating of target scenarios and the mean rating of the anchor-generating scenario for the high and low anchor conditions from the calibration group are presented in Table A2

Table S2. Mean emotion intensity rating of younger and older calibration groups

	Target scenarios	High anchor scenarios	Low anchor scenarios
Older adults	67.14±11.48	82.11±9.85	62.02±13.03
Younger adults	66.81±11.36	77.64±10.81	60.13±12.58

Supplement B

The detailed process of baseline task

The baseline task contained only the judgment phase emotion estimates of the

protagonist in scenarios without any anchor or comparison phase. As a result, the performance difference in the experimental task between the high- and low-anchor groups could be attributed to the anchoring effect. The baseline task in the two experiments all contained 5 trials (one trial for each emotion).

The baseline task verified that no significant difference existed in judgment tendency between the two anchor groups. T-test results showed no significant difference between the mean estimates of the two anchor groups, M_{high}=69.98; M_{low}=70.54， $t(126)$=0.254, p=.800, MD=0.59. Therefore, the confounding effect of judgment tendency could be excluded.

Supplement C

The Pearson correlation matrices

Table S3. Correlation matrices between variables in present study for older adults

	1 Anchor	2 Age	3 Gender	4 Education	5 E_Joy	6 E_Pride	7 E_Anger	8 E_Distress	9 E_Sadness	10 PS	11 WM	12 Inhibition
1	1											
2	0.009	1										
3	-0.269*	-0.333**	1									
4	-0.043	-0.02	0.005	1								
5	-0.296*	-0.045	0.165	-0.112	1							
6	-0.394**	-0.144	0.298*	-0.112	0.644**	1						
7	-0.214	-0.007	0.348**	0.001	0.253*	0.433**	1					
8	-0.354**	-0.202	0.378**	0.101	0.282*	0.493**	0.689**	1				
9	-0.309*	-0.045	0.345**	-0.041	0.438**	0.414**	0.544**	0.609**	1			
10	0.09	-0.304*	0.176	0.179	0.118	-0.01	0.008	0.195	0.082	1		
11	0.022	-0.051	-0.153	0.272*	0.042	0.02	-0.005	0.098	0.004	0.108	1	
12	-0.197	-0.122	0.14	-0.077	-0.012	0.055	0.007	0.078	-0.012	-0.015	-0.192	1

Note: * Correlation is significant at the 0.05 level (2-tailed). ** Correlation is significant at the 0.01 level (2-tailed). Anchor means anchor condition (0=high anchor, 1=low anchor). E_Joy means participants' mean estimates in judging joy. PS refers to processing speed. WM refers to working memory.

Table S4. Correlation matrices between variables in present study for younger adults

	1 Anchor	2 Age	3 Gender	4 Education	5 E_Joy	6 E_Pride	7 E_Anger	8 E_Distress	9 E_Sadness	10 PS	11 WM	12 Inhibition
1	1											
2	-0.062	1										
3	0.076	-0.118	1									
4	-0.194	0.659**	0.035	1								
5	-0.189	0.213	-0.021	0.14	1							
6	-0.259*	0.134	0.157	0.223	0.530**	1						
7	-0.260*	0.038	0.138	0.208	0.397**	0.614**	1					
8	-0.053	-0.074	0.287*	0.168	0.257*	0.401**	0.591**	1				
9	-0.23	-0.007	0.084	0.238	0.442**	0.455**	0.612**	0.578**	1			
10	0.023	-0.281*	0.295*	0.065	-0.008	0.036	0.168	0.310*	0.227	1		

11	0.107	-0.343**	0.064	0.008	-0.108	0.004	0.086	0.152	0.049	0.364**	1	
12	-0.103	0.03	-0.127	-0.013	0.136	0.133	0.084	0.089	0.095	-0.032	0.047	1

Supplement D

The detailed process of ANCOVA

As we tried to analyze the anchoring effect and corresponding effects for younger adults and older adults, years of education should be considered as covariate in analysis, because of its' significant difference in two age groups. However, Schneider et al., (2015) suggested ANCOVAs results could yield bias estimates when the mean values of the covariates differ across the two age groups, in mixed design. They recommend to conduct a standard ANCOVA to test the effect of covariate and the Within∗Covariate interaction, but a standard repeated measures ANOVA (without the covariate) is needed to evaluate all other effects. Thus, at first, 2 (age: young, old) × 2 (anchor: high, low) × 5 (emotional category: joy, pride, anger, distress, sadness) repeated measures ANCOVA was conducted on the mean estimates, with centered years of education as covariate. The results indicated that, the main effect of education was not significant, $F\ (1,118) = 1.29$, $p = 0.706$, $\eta_p^2 = 0.001$, and the interaction effect between education and emotion category was also not significant, $F\ (1,118) = 0.02$, $p = 0.896$, $\eta_p^2 = 0$.
